# *Pogostemon puerensis* (Lamiaceae), a new species from Yunnan of China

**DOI:** 10.3897/phytokeys.274.186914

**Published:** 2026-05-04

**Authors:** Xue-Xue Wu, Chao Xu, Zhi-Hong Li, Yan-Yi Chen, Hai-Lei Zheng, Qiang Wang

**Affiliations:** 1 State Key Laboratory of Plant Diversity and Specialty Crops & State Key Laboratory of Systematic and Evolutionary Botany, Institute of Botany, Chinese Academy of Sciences, Beijing 100093, China National Botanical Garden, Institute of Botany, Chinese Academy of Sciences Beijing China; 2 National Botanical Garden, Institute of Botany, Chinese Academy of Sciences, Beijing 100093, China State Key Laboratory of Plant Diversity and Specialty Crops & State Key Laboratory of Systematic and Evolutionary Botany, Institute of Botany, Chinese Academy of Sciences Beijing China; 3 College of Life Sciences, University of Chinese Academy of Sciences, Beijing 100049, China College of Life Sciences, University of Chinese Academy of Sciences Beijing China; 4 Administration Bureau of Sun-River Provincial Nature Reserve, Simao, Yunnan 665099, China Administration Bureau of Sun-River Provincial Nature Reserve Yunnan China

**Keywords:** Morphology, new species, Pogostemoneae, *
Pogostemon
*, taxonomy

## Abstract

*Pogostemon* is the largest genus in tribe Pogostemoneae (Lamiaceae) and exhibits considerable morphological diversity, which has complicated species delimitation in parts of Southeast Asia. During botanical surveys in Pu’er City, Yunnan Province, China, a distinctive population of *Pogostemon* resembling *P. hainanensis* and *P. parviflorus* was discovered but differs in several stable morphological characters. Its taxonomic status was assessed using comparative morphology and molecular phylogenetic analyses based on five plastid regions (*matK*, *psbA–trnH*, *rbcL*, *rps16*, *trnL–trnF*) and the nrITS region. The sampling included 57 accessions representing 35 species of *Pogostemon*, with representatives of other genera in Pogostemoneae and related tribes as outgroups. Bayesian and maximum likelihood analyses recovered the Pu’er plant as a well-supported lineage within *Pogostemon*, sister to *P. parviflorus* in the plastid dataset and to *P. cablin* in the nuclear dataset. Morphologically, it differs from *P. hainanensis*, *P. parviflorus*, and *P. cablin* by its much shorter bracteoles, distinctly exserted corolla, and longer stigma lobes; it is further distinguished from *P. hainanensis* and *P. parviflorus* by the densely hairy upper two-thirds of the inner calyx tube and from *P. cablin* by its different phenology. It is therefore described as a new species, *Pogostemon puerensis* X.X.Wu, Z.H.Li & Qiang Wang.

## Introduction

Lamiaceae is among the six largest angiosperm families and is widely recognized for its high diversity of aromatic taxa rich in essential oils, many of which are economically important as ornamentals (e.g., *Salvia* L. and *Nepeta* L.), culinary herbs (e.g., *Thymus* L., *Mentha* L., and *Ocimum* L.), and sources of fragrance ingredients (e.g., *Lavandula* L.). Recent plastome phylogenomics has provided the most comprehensive tribal framework to date, supporting the monophyly of 12 subfamilies and recognizing 22 tribes (Zhao et al. 2021a). Lamioideae is among the most species-rich subfamilies, and recent classifications recognize 13 tribes within the subfamily (Scheen et al. 2010; Bendiksby et al. 2011; Roy and Lindqvist 2015; Li et al. 2016; Siadati et al. 2018; Zhao et al. 2021b; Qian et al. 2024; Surina et al. 2025). Pogostemoneae Briq. is repeatedly recovered as an early-diverging lineage within Lamioideae (Scheen et al. 2010; Li et al. 2016; Zhao et al. 2021a, 2021b). Traditionally, the tribe has been circumscribed to include approximately 13 genera, many of which are monotypic or species-poor (Wu et al. 2025).

*Pogostemon* Desf. is the largest genus of Pogostemoneae and currently comprises 93 accepted species distributed in tropical and subtropical Asia, central Africa, and northern Oceania (POWO 2025). A prominent example of its economic relevance is patchouli, *Pogostemon cablin* (Blanco) Benth., whose leaves and aerial parts are distilled to produce patchouli oil, a widely used base note and fixative in perfumery and related fragrance products. However, the circumscription of *Pogostemon* has historically been contentious, primarily concerning whether the genus should be narrowly defined (*Pogostemon* sensu stricto) or broadly delimited (*Pogostemon* sensu lato) to include *Dysophylla* Blume.

Desfontaines (1815) established the genus *Pogostemon* based on the species *Pogostemon plectranthoides* Desf., highlighting bead-like hairs on the filaments as a critical diagnostic character. Subsequently, Blume (1826) described the genus *Dysophylla* based on *Dysophylla auricularia* (L.) Blume, characterized by opposite leaves, a calyx closed at the fruiting stage, a fleshy, swollen disc, and similarly bead-like hairs on filaments. Later, *Dysophylla* expanded to include taxa with verticillate leaf arrangements, which were once proposed as a separate genus, *Eusteralis* Raf. (Rafinesque 1837), but this proposal was not widely accepted.

Given the shared morphological feature of bead-like hairs on the filaments, Hasskarl (1842) suggested merging *Dysophylla* into *Pogostemon*, thus proposing a broader concept of *Pogostemon* (*Pogostemon* s.l.). This broad concept has received robust support from molecular phylogenetic studies initiated by Bendiksby et al. (2011) and reinforced by subsequent comprehensive sampling studies. Currently, *Pogostemon* s.l., incorporating traditional *Pogostemon* s.str. and *Dysophylla*, is widely accepted (Yao et al. 2016; Hu et al. 2021; Zhao et al. 2021a, 2021b; Tan and Yao 2024; Wu et al. 2025).

Due to significant morphological variation within *Pogostemon* s.l., differing views persist regarding the relationships among its constituent species (Bentham 1833; Briquet 1897; Kudo 1929). Recently, Yao et al. (2016) provided an updated infrageneric classification based on molecular phylogenetics and trait evolution analyses, dividing the genus into two subgenera: subgen. *Pogostemon*, comprising species from sect. *Verticillati* (sensu stricto), and subgen. *Dysophyllus*, including all traditional *Dysophylla* species and sect. *Racemosi* species from *Pogostemon* s.str. Additionally, a recent comprehensive review indicated that China harbors 29 species and two varieties of *Pogostemon* (Tan and Yao 2024).

During a biodiversity survey conducted in Pu’er, Yunnan Province, one of the authors discovered a *Pogostemon* species morphologically similar to the recently described Hainan endemic *Pogostemon hainanensis* L.X. Yuan & Gang Yao and *Pogostemon parviflorus* Benth., known from Hong Kong and Guangzhou. Initially, the possibility that this represented a newly reported distribution of an existing species was considered. However, after detailed field examinations carried out by the team in 2025, clear morphological distinctions were identified between this plant and both *P. hainanensis* and *P. parviflorus*, particularly in bracteole length, indumentum on the inner surface of the calyx tube, and corolla structure and coloration. Additionally, the phylogenetic relationships were reconstructed by incorporating five chloroplast DNA regions and the ITS region of the newly discovered plant along with available global taxa from the tribe Pogostemoneae in public databases. Based on both morphological differentiation and molecular phylogenetic evidence, this plant from Pu’er, Yunnan, is described as a new species of *Pogostemon*.

## Materials and methods

### Phylogenetic study

To assess the phylogenetic position of the new species within *Pogostemon*, phylogenetic analysis was conducted based on five plastid regions (*matK*, *psbA–trnH*, *rbcL*, *rps16*, *trnL–trnF*) and the nuclear ribosomal ITS (nrITS) region, broadly following Yao et al. (2016) and Yuan et al. (2022). Total genomic DNA of the new species was extracted from silica-gel–dried leaves (voucher specimens WXX25015 and LZH25001, PE) following a modified CTAB protocol of Li et al. (2013). Primers for *matK* and *rps16* loci were newly designed for amplification in this study, while some universal primers were employed for the remaining DNA regions. Details of all primers are provided in Suppl. material 1: table SS1. PCR amplifications were carried out under standard three-step cycling conditions. The resulting products were sequenced unidirectionally. Raw Sanger sequences were verified for accuracy using Sequencher 5.4.5. Sequences for the remaining taxa were taken from Yuan et al. (2022) and newly downloaded from GenBank (NCBI, www.ncbi.nlm.nih.gov).

In total, 57 accessions representing 35 species of *Pogostemon* were included, comprising 34 accessions of 16 species of subg. *Pogostemon* and 23 accessions of 19 species of subg. *Dysophyllus*. Representatives of the other nine genera of Pogostemoneae, together with *Gomphostemma* Wall. ex Benth. (Gomphostemmateae) and *Colquhounia* Wall. (Colquhounieae) were used as outgroups following the phylogenetic framework of Yao et al. (2016) and Zhao et al. (2021b). Detailed voucher and sequence information for all sampled taxa, including the newly generated sequences of the new species, is provided in Suppl. material 1: table S2.

All sequences were aligned with MAFFT v7.221 (Katoh and Standley 2013). Two datasets were thus constructed: (1) a cpDNA dataset comprising the five plastid regions (*matK*, *psbA–trnH*, *rbcL*, *rps16*, and *trnL–trnF*) and (2) the nrITS dataset. Given the pronounced nuclear–plastid discordance known in *Pogostemon* and the incongruence between the plastid and nuclear phylogenies recovered here, previous studies were not followed in concatenating plastid and nuclear data into a single combined dataset for analysis (Yao et al. 2016; Yuan et al. 2022). Phylogenetic analyses were conducted using Bayesian inference (BI) and maximum likelihood (ML). BI analyses were performed in MrBayes v3.2.7a (Ronquist et al. 2012) using four Markov chains run for 1,000,000 generations, sampling every 1,000 generations, and discarding the first 25% of trees as burn-in. Convergence was assessed by effective sample sizes (ESS > 200) and an average standard deviation of split frequencies < 0.01. ML analyses were carried out in RAxML v8.2.12 (Stamatakis 2014) under the GTRGAMMA model with 1,000 bootstrap replicates.

### Morphological study

Morphological variation among *Pogostemon* taxa was assessed using online images of herbarium specimens from BR (Meise Botanic Garden), CANB (Australian National Herbarium), E (Royal Botanic Garden Edinburgh), K (Royal Botanic Gardens, Kew), MICH (University of Michigan Herbarium), P (Muséum national d’Histoire naturelle), L/U/WAG (Naturalis Biodiversity Center), and PE (Institute of Botany, Chinese Academy of Sciences), together with original protologues and a subset of specimens examined in hand. Photographs of the new species were taken with an Olympus OM-D EM-5 Mark III and a Nikon D7500 digital camera. Fine structural features were examined and documented under a Leica M205 C stereomicroscope.

## Results and discussion

### Phylogenetic analyses

**Figure 1.**
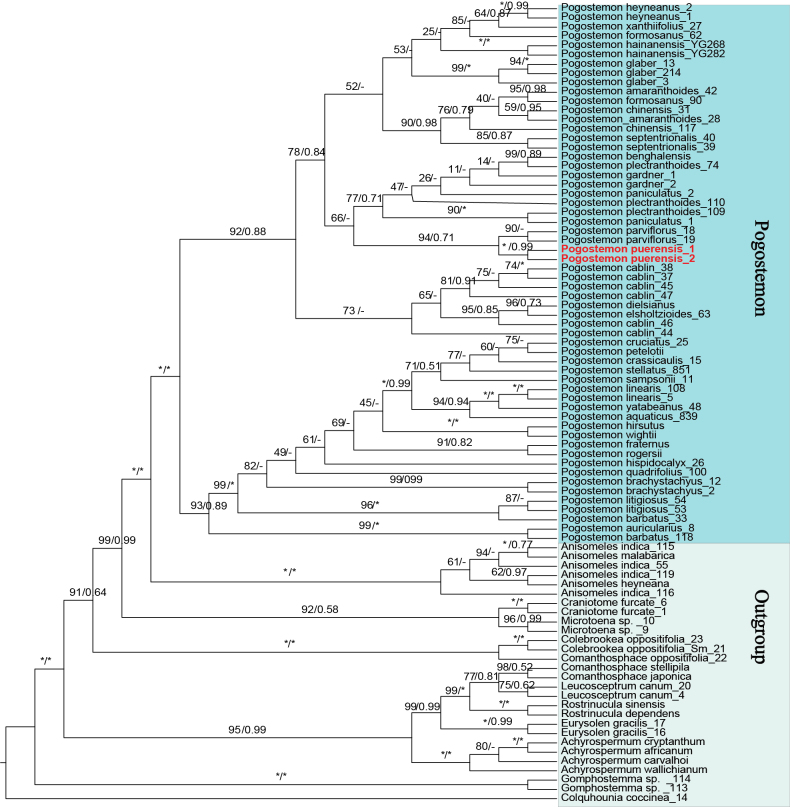
Phylogenies of *Pogostemon* and its relatives based on the cpDNA dataset (including *matK*, *psbA–trnH*, *rbcL*, *rps16*, and *trnL–trnF*) using a concatenated approach, including ML and BI methods. Numbers above branches indicate ML bootstrap (BS) / BI posterior probability (PP) support values. “*” indicates maximal support and “–” marks topological incongruence.

**Figure 2.**
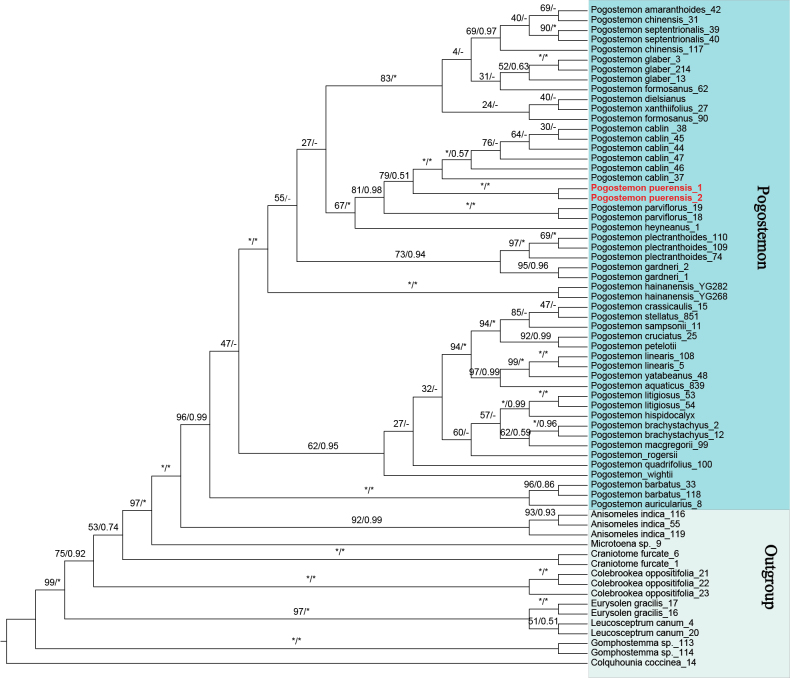
Phylogenies of *Pogostemon* and its relatives based on the nrITS dataset using a concatenated approach, including ML and BI methods. Numbers above branches indicate ML bootstrap (BS) / BI posterior probability (PP) support value. “*” indicates maximal support and “–” marks topological incongruence.

The cpDNA and the nrITS datasets were 2525 bp and 618 bp in length, respectively. The two datasets recovered broadly congruent topologies for *Pogostemon*, with differences restricted to a few weakly supported nodes (defined as BS < 80% or PP < 0.80) (Figs 1, 2). Both datasets supported the monophyly of *Pogostemon* and recovered it as a sister to *Anisomeles* R.Br. However, the two datasets differed somewhat in the inferred relationships within *Pogostemon*. In the cpDNA analyses, *Pogostemon* comprised two strongly supported clades corresponding to subg. *Pogostemon* (BS/PP = 92% / 0.88) and subg. *Dysophyllus* (BS/PP = 93% / 0.89) (Fig. 1). This overall topology agrees with earlier studies (Yao et al. 2016; Wu et al. 2025). In contrast, the nrITS tree resolved three clades within *Pogostemon*, with subg. *Dysophyllus* split into two weakly supported groups (Fig. 2). This pattern matches the nrITS-based topology reported by Yuan et al. (2022), although it was not discussed there. One possible explanation is that many nrITS sequences in public databases contain long homopolymeric poly-A/T tracts, which can reduce alignment quality and weaken tree inference. To reduce analytical complexity and minimize potential artefacts caused by excessive outgroup sampling, the nrITS-based phylogenetic analysis was repeated using only representatives of *Anisomeles* as the outgroup (Suppl. material 2: fig. S1). The resulting topology was essentially consistent with that obtained from the full ITS dataset, again recovering three clades. Minor differences were confined to the positions of a few weakly supported species within clades. Therefore, the simplified analysis is not discussed further (Fig. 2; Suppl. material 2: fig. S1). Notably, the results indicate marked cytonuclear discordance within *Pogostemon*, and infrageneric relationships in the genus will require further study based on expanded plastid and nuclear datasets.

Across all datasets, accessions of the new species formed a well-supported monophyletic group nested within subg. *Pogostemon*. However, its placement within subg. *Pogostemon* differed among datasets. In the cpDNA analyses, the new species was recovered as sister to *P. parviflorus* (BS/PP = 94% / 0.71; Fig. 1). In the nrITS analysis, it was sister to *P. cablin* (BS/PP = 79% / 0.51; Fig. 2), and this pair was in turn sister to *P. parviflorus* (BS/PP = 81% / 0.98; Fig. 2). The new species is resolved as a sister to different taxa in the two datasets. Morphologically, it shows similarities to both putative sister taxa but can nevertheless be readily distinguished from each of them. These phylogenetic relationships and morphological differences are discussed below.

At present, 93 species of *Pogostemon* are recognized worldwide, including 29 species and two varieties in China (Yao et al. 2015; Liu et al. 2021; Yuan et al. 2022; Tan and Yao 2024). Because of the rarity of material and the unavailability of suitable DNA for sequencing, the phylogenetic analyses did not include *P. monticola* T.C. Hsu & S.W. Chung and 12 other species that have been assigned to subg. *Pogostemon*, such as *P. nelsonii* Doan, *P. nepetoides* Stapf, *P. pubescens* Benth., *P. purpurascens* Dalzell, *P. tuberculosus* Benth., *P. villosus* Benth., and *P. wattii* C.B. Clarke. Although unsampled in the molecular analyses, these species differ from the new species from Yunnan in several morphological characters, which are discussed in detail below.

### Morphological comparison

**Figure 3.**
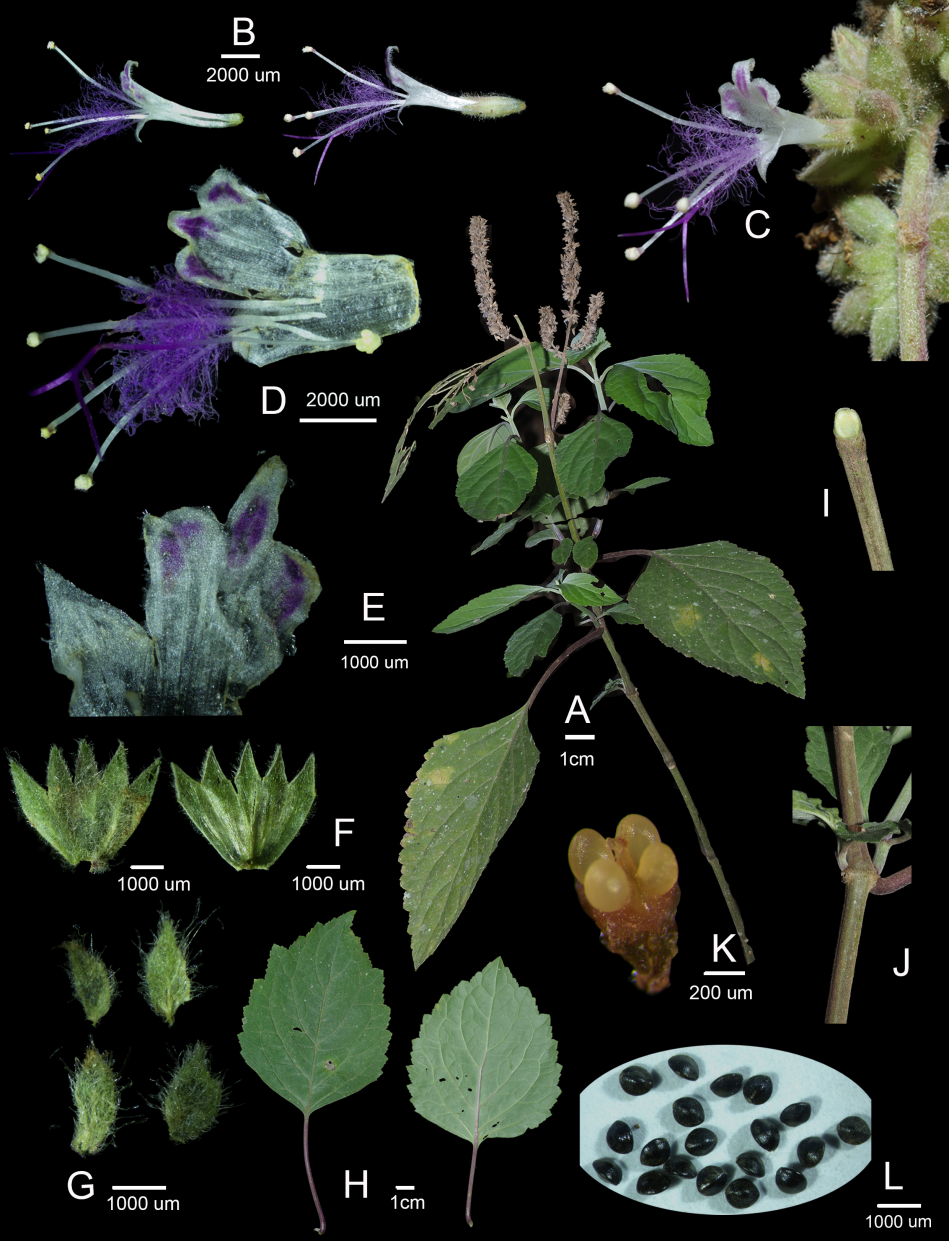
Images of *Pogostemon puerensis* X.X.Wu, Z.H.Li & Qiang Wang, sp. nov. **A**. Individual in the fruiting period of wild populations; **B**. Corolla and corolla with calyx; **C**. Partial inflorescence in lateral view; **D, E**. Dissected corolla; **F**. Dissected calyces (outside and inside); **G**. Bracteoles; **H**. Leaves (adaxial and abaxial); **I, J**. Stem; **K**. Nutlets (immature); **L**. Nutlets (mature).

The new species described here (Fig. 3A–L) can be readily assigned to *Pogostemon* based on the conspicuous beard of hairs at the middle of the filaments and the small, smooth nutlets (Fig. 3B–D, L). It is further placed in *Pogostemon* subg. *Pogostemon* by its paniculate inflorescences formed by spikes with lateral branches (Fig. 3A), the solid and distinctly quadrangular stems (Fig. 3I, J), and the bracts with conspicuous midvein and lateral veins (Fig. 3G). This placement is also supported by the molecular phylogenetic analyses (Figs 1, 2).

Morphologically, the new species is similar to *Pogostemon hainanensis* in having quadrangular stems, leaves with conspicuously double-serrate margins, a slightly inflated tubular calyx, and a comparable calyx length. It also shares several traits with *P. parviflorus*, including similar plant height, double-serrate leaves, ovate-lanceolate to ovate bracts and bracteoles, and a slightly inflated tubular calyx. In plastid phylogenetic analyses, the new species is recovered as the strongly supported sister to *P. parviflorus* (Fig. 1). Despite these similarities, the new species can be clearly distinguished from both taxa by a combination of characters, particularly leaf venation, inflorescence length, bracteole length, and the degree of corolla exsertion. Leaf venation provides an additional diagnostic character: the new species typically has five pairs of lateral veins, occasionally three or four, whereas *P. hainanensis* usually has three to four pairs and only rarely five. Inflorescence architecture is also informative. The sister species *P. parviflorus* has much shorter spikes (0.7–3.5 cm), whereas those of the new species are considerably longer, ca. 4.5–10 cm. Bracteoles in the new species are markedly shorter (ca. 1 mm) than those of *P. parviflorus* (4–6 mm) and *P. hainanensis* (2.5–5 mm). Calyx morphology and indumentum, together with the corolla-to-calyx length ratio, further differentiate the new species. Its calyx is tubular and slightly inflated, ca. 4 mm long, with the inner surface densely hairy in the upper two-thirds. The corolla is distinctly exserted, extending beyond the calyx by approximately 1.5 times its length. In contrast, the corolla in both *P. parviflorus* and *P. hainanensis* is only slightly exserted, and the inner surface of the calyx tube is sparsely hairy or nearly glabrous. The main diagnostic characters are summarized in Table 1.

**Table 1.** Diagnostic morphological comparison highlighting key distinguishing characters among *Pogostemon puerensis* X.X.Wu, Z.H.Li & Qiang Wang, *P. hainanensis* L.X. Yuan & Gang Yao, *P. parviflorus* Bentham, and *P. cablin* (Blanco) Benth.

Morphology	*P. puerensis*	*P. hainanensis*	*P. parviflorus*	*P. cablin*
Habit	perennial herb or subshrub	perennial herb or subshrub	perennial herb or subshrub	perennial herb or subshrub, often cultivated
Plant height (m)	0.3–1.5	0.8–2.0	0.3–0.6	up to 1.5
Leaf lateral veins (pairs)	5 (rarely 3)	3–4 (rarely 5)	4–5	4–5
Bracteole length (mm)	1.0–2.0	2.5–5.0	4.0–6.0	4.0–8.0
Inner surface of the calyx tube	densely hairy on upper 2/3 of tube	occasionally hairy near upper part of tube	sparsely strigillose on upper part of tube	sparsely puberulent inside tube
Corolla color	white with purplish-red blotches on middle lobe of upper lip	entirely white	white, pink, or purplish	white or slightly tinged; upper lip spotted
Corolla exsertion	strongly exserted (1.0–1.5× calyx)	slightly exserted	slightly exserted	slightly exserted
Inner surface of corolla tube	with a conspicuous ring of hairs	without a conspicuous ring of hairs	without a conspicuous ring of hairs	not recorded
Length of stigma lobes (mm)	2.8–3.1	1.2–1.7	0.7	2.5–3.0
Phenology	Fl. Dec–Feb; Fr. Jan–May	Fl. Dec–Feb; Fr. Jan–Apr	Fl. Aug–Nov; Fr. Oct–Dec	Fl. Mar–May

The corolla of the new species is distinctly bilabiate, with the typical 3/1 division of lobes (Fig. 3B–E), a common feature in many species of *Pogostemon* subg. *Pogostemon*. However, the new species is remarkable in having regular purplish-red markings on all three lobes of the upper lip (Fig. 3C–E). This character was consistently observed in all wild populations examined, indicating that it is stable under natural conditions. Previous descriptions of corolla color in *Pogostemon* have often been generalized as white, pink, or pale purple. However, based on field observations and inspection of color photographs available from the Plant Photo Bank of China (PPBC; https://ppbc.iplant.cn/; accessed 3 March 2026) and the Chinese Field Herbarium (CFH; http://139.196.237.66:8088/; accessed 3 March 2026), corolla color in the genus is more variable than previously recognized and is not necessarily uniform. For example, *P. dielsianus* Dunn (Hu et al. 2021), *P. elsholtzioides* Benth., and *P. brachystachyus* Benth. show a similar rose tone, often grading from the corolla limb to the tube, toward paler hues. In *P. menthoides* Blume, the corolla is largely white, with pink restricted to the margins of the upper and lower lips, whereas the corolla is uniformly white in *P. hainanensis*, *P. monticola* (Liu et al. 2021), and some individuals of *P. parviflorus*. To current knowledge, the condition seen in *P. puerensis*, with discrete color patches on the upper lip, is relatively uncommon. In the present study, the phylogenetic tree inferred from nuclear markers recovered the new species and *P. cablin* as sister taxa. However, the two differ markedly in phenology and morphology. Nevertheless, according to online images and original literature (Bentham 1848), the cultivated species *P. cablin* shows a somewhat similar corolla pattern to that of the new species, namely a white or slightly tinged corolla with spotted markings on the upper lip (Table 1). These patterns of corolla color and patch distribution may provide useful diagnostic characters in *Pogostemon*, and future species descriptions should record corolla color in greater detail.

The stigma lobes are conspicuously long in the new species, reaching up to 3.1 mm (Fig. 3B–D). Within *Pogostemon*, similarly long stigma lobes have also been reported in *P. griffithii* (ca. 2.5 mm), *P. latifolius* (2.0–2.5 mm; treated at species rank by Yao), and *P. cablin* (2.5–3.0 mm), the latter of which is recovered as a sister to the new species in the ITS phylogeny. By contrast, the two morphologically similar species, *P. hainanensis* and *P. parviflorus*, have much shorter stigma lobes, measuring 1.2–1.7 mm and ca. 0.7 mm, respectively. Nevertheless, *P. griffithii* and *P. latifolius* differ markedly from the new species in several other morphological characters, including plant height, leaf morphology, petiole length, and corolla color, while *P. cablin* can be readily distinguished from the new species by differences in phenology and bracteole length.

In China, *Pogostemon* is currently represented by 29 species and two varieties. In Pu’er City, Yunnan, the only species of subg. *Pogostemon* previously recorded is *P. glaber* Benth. In Yunnan Province, additional members of subg. *Pogostemon* include *P. chinensis* C.Y. Wu & Y.C. Huang, *P. amaranthoides* Benth., *Pogostemon glaber* var. *tsingpingensis* (C. Y. Wu & Y. C. Huang) G. Yao, *Pogostemon latifolius* (C. Y. Wu & Y. C. Huang) G. Yao, and *Pogostemon dielsianus* Dunn. Among these, *P. glaber* var. *tsingpingensis* bears some superficial resemblance to the new species, but it can be readily distinguished by the long, soft hairs on the stems and leaves, as well as on the outer surface of the calyx. The remaining species in Yunnan differ clearly from *P. puerensis* in habit, plant size, leaf morphology, corolla color, degree of corolla exsertion, and the size and shape of bracteoles. In Pu’er City, subg. *Dysophyllus* is represented by *P. brachystachyus* Benth. and *P. quadrifolius* (Benth.) F. Muell., and field surveys also confirmed the occurrence of *P. fraternus* Miq. Many additional species of subg. *Dysophyllus* occur in Yunnan. As the new species belongs to subg. *Pogostemon* and is morphologically distant from these taxa, they are not discussed further here. A detailed morphological comparison of the new species with its putative relatives and morphologically similar species is provided in Table 1. Thus, the combined morphological and molecular phylogenetic evidence supports the recognition of the Yunnan material as a new species of *Pogostemon*, here described as *Pogostemon puerensis* X.X.Wu, Z.H.Li & Qiang Wang.

### Taxonomic treatment

#### Pogostemon puerensis

Taxon classification

Plantae

LamialesLamiaceae

X.X.Wu, Z.H.Li & Qiang Wang sp. nov.

F5A54079-3DC1-5016-98D5-7CD2E973749B

urn:lsid:ipni.org:names:77378698-1

Figs 3, 4

##### Type.

China • Yunnan province, Pu’er City, Ning’er Hani and Yi Autonomous County, Ning’er town, along County Road X013, ca. 1.4 km N of Xiaoheijiang Forest Park, at an elevation of approximately 933 m, 9 May 2025, *X.X. Wu et al. WXX25015* (holotype, PE; isotype, PE).

**Figure 4.**
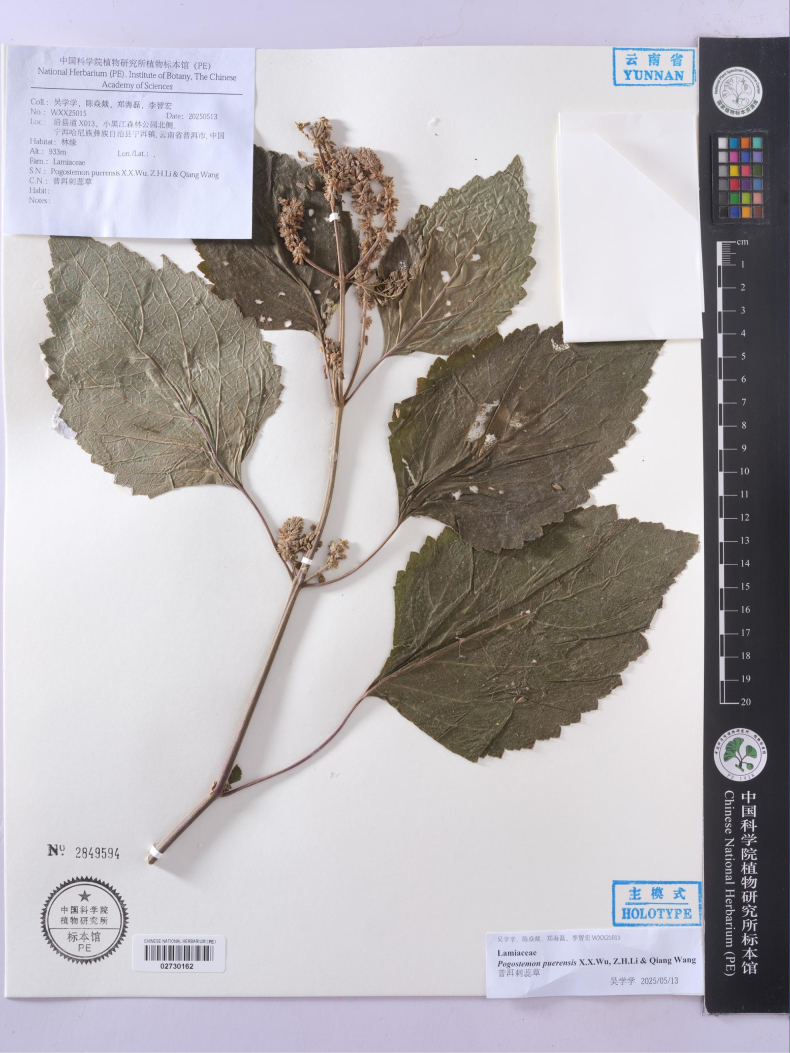
Holotype of *Pogostemon puerensis* X.X.Wu, Z.H.Li & Qiang Wang, sp. nov.

##### Diagnosis.

*Pogostemon puerensis* is morphologically similar to *P. hainanensis* and *P. parviflorus* but can be readily distinguished from both by its plant height 0.3–1.5 m (vs. 0.8–2 m in *P. hainanensis* and 0.3–0.6 m in *P. parviflorus*); lateral veins five pairs on each side of the midvein (vs. 3–4 pairs in *P. hainanensis* and 4–5 pairs in *P. parviflorus*); bracteoles 1–2 mm long (vs. 2.5–5 mm in *P. hainanensis* and 4–6 mm in *P. parviflorus*); the inner surface of the calyx tube densely hairy in the upper two-thirds (vs. occasionally hairy only near the upper part of the tube in *P. hainanensis* and sparsely strigillose only in the upper part of the tube in *P. parviflorus*); corolla with purplish-red blotches on the middle lobe of the 3-lobed upper lip (vs. corolla entirely white in *P. hainanensis* and white, pink, or purplish in *P. parviflorus*); corolla tube with a conspicuous ring of hairs (vs. without a conspicuous ring of hairs); and stigma lobes 2.8–3.1 mm long (vs. 1.2–1.7 mm in *P. hainanensis* and ca. 0.7 mm in *P. parviflorus*).

The new species can be further distinguished from its sister species in the ITS phylogeny, *P. cablin*, by its shorter bracteoles (1–2 mm vs. 4–8 mm), strongly exserted corolla (1.0–1.5× calyx length vs. slightly exserted), and differences in phenology (flowering from December to February vs. March to May).

##### Description.

Perennial herbs; base woody; plants ca. 0.3–1.5 m tall. Stems erect, 3–5 mm in diam., quadrangular, solid; nodes slightly swollen; unbranched or sparsely branched; stems densely pubescent with soft hairs. Leaves opposite, papery to membranous; blade ovate to ovate-elliptic, 7.5–14.1(–16.0) × 5.4–9.31(–10.2) cm; apex attenuate; base cuneate to broadly cuneate; margin coarsely serrate except entire near base, teeth obtuse-rounded or acute; midrib prominent abaxially; lateral veins 5(rarely 3) pairs, raised on abaxial surface; both surfaces densely covered with ferrugineous strigose hairs. Petiole (–3.7)4.22–6.66(–7.5) cm long. Inflorescences verticillasters arranged in a simple, continuous spiciform inflorescence, (1.0–)4.5–10 cm long, terminal or axillary; peduncle 0.5–3(–4.5) cm long, densely ferrugineous-pubescent; verticillasters becoming more widely spaced toward the base at anthesis, appearing interrupted, this feature inconspicuous in fruit; verticillasters many-flowered; flowers sessile. Bracteoles ovate-lanceolate to ovate-orbicular, 1–2 mm long, conspicuously shorter than calyx, with 1–2 pairs of lateral veins, densely long-pubescent. Calyx tubular, inflated, ca. 4 mm long, 5-veined; externally shortly pubescent; inner surface pubescent in upper 2/3, glabrous below; teeth narrowly triangular, equal, ca. 1/2 as long as calyx tube. Corolla white, ca. 8–9 mm long, exserted 1–1.5 × length of calyx; bilabiate; upper lip 3-lobed, each lobe marked with purplish-red spots; margins of outer lobes sparsely pubescent, otherwise glabrous; upper lip 6.5–7 mm long; lower lip entire. Stamens 4, erect, strongly exserted; filaments 7–7.5 mm long, inserted ca. 2 mm above base of corolla tube, bearded at middle, beard hairs largely exserted; corolla tube with a fascicle of hairs at point of filament insertion; anthers 1-locellate, dehiscing apically. Style 7–7.5 mm long; stigma 2-lobed, lobes subequal, 2.8–3.1 mm long. Disc ca. 0.6 mm long. Nutlets 4, smooth, black, glossy, ca. 0.7 × 0.6 mm, ovate to slightly depressed-globose.

##### Distribution and habitat.

**Figure 5.**
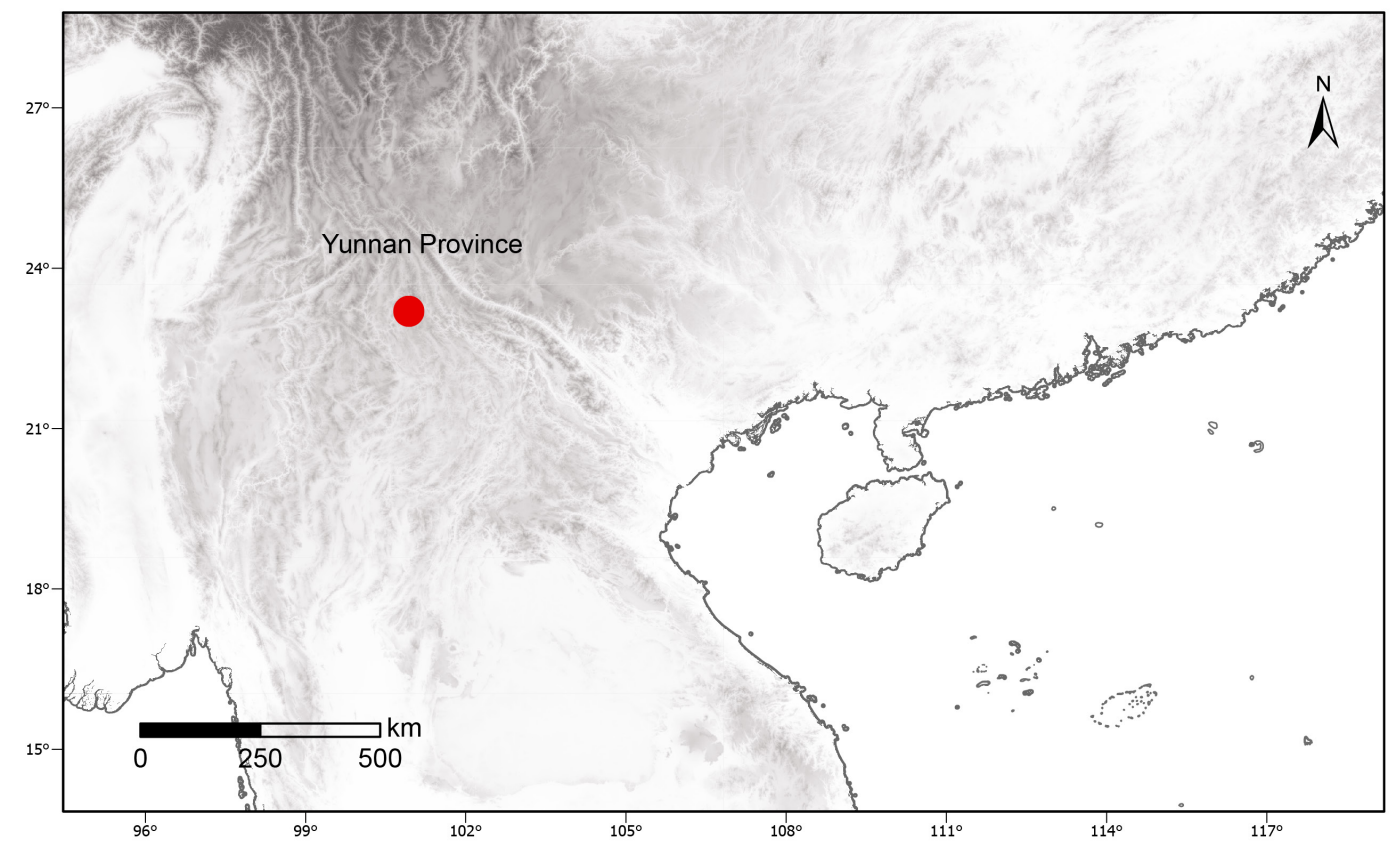
Distribution of *Pogostemon puerensis* (red circular).

The new species is currently known only from the vicinity of the type locality in Pu’er, Yunnan, China (Fig. 5). It grows in the forest understory near a deep, water-filled gully, with individuals scattered along the gully margin, the only known population of *Pogostemon puerensis* comprises approximately 30 individuals.

##### Etymology.

*Pogostemon puerensis* is named after its type locality, Yunnan province, Pu'er City, China.

##### Phenology.

Flowering from December to February of the following year and fruiting from January to May.

##### Vernacular name.

Simplified Chinese: 普洱刺蕊草 (Chinese pinyin: pŭ ě cì ruĭ căo).

## Supplementary Material

XML Treatment for Pogostemon puerensis

## Additional information

### Artificial Intelligence (AI) use

The authors accept full responsibility for the content of the manuscript, including the disclosure of any use of AI.

No AI tools were used in the preparation of this manuscript.

### Author ORCIDs

X.-X. Wu https://orcid.org/0009-0006-8645-4100

C. Xu https://orcid.org/0000-0002-9678-4772

Z.-H. Li https://orcid.org/0000-0002-5198-1502

Y.-Y. Chen https://orcid.org/0009-0008-5864-8121

H.-L. Zheng https://orcid.org/0000-0002-3774-7453

Q. Wang https://orcid.org/0000-0002-0699-2034

### Data availability

All data that support the findings of this study are deposited in GenBank.

## Supplementary materials

Supplementary material 1 Supplementary tables Data type xlsx Explanation note **table SS1**. Primers used for PCR amplification and Sanger sequencing of six DNA regions in *Pogostemon* (nrITS, *matK*, *psbA–trnH*, *rbcL*, *rps16*, *trnL–trnF*), including newly designed primers; **table S2**. Voucher information and GenBank accession numbers for taxa sampled in this study, including *Pogostemon* and outgroups used in the phylogenetic analyses. https://binary.pensoft.net/file/1623433This dataset is made available under the Open Database License (http://opendatacommons.org/licenses/odbl/1.0/). The Open Database License (ODbL) is a license agreement intended to allow users to freely share, modify, and use this Dataset while maintaining this same freedom for others, provided that the original source and author(s) are credited. Xue-Xue Wu, Chao Xu, Zhi-Hong Li, Yan-Yi Chen, Hai-Lei Zheng, Qiang Wang

Supplementary material 2 Phylogenies of *Pogostemon* and related taxa based on the nrITS dataset, with *Anisomeles* as the sole outgroup, inferred using concatenated ML and BI analyses Data type pdf Explanation note Numbers above branches indicate ML bootstrap (BS) / BI posterior probability (PP) support values. “*” indicates maximal support and “–” marks topological incongruence. https://binary.pensoft.net/file/1623434This dataset is made available under the Open Database License (http://opendatacommons.org/licenses/odbl/1.0/). The Open Database License (ODbL) is a license agreement intended to allow users to freely share, modify, and use this Dataset while maintaining this same freedom for others, provided that the original source and author(s) are credited. Xue-Xue Wu, Chao Xu, Zhi-Hong Li, Yan-Yi Chen, Hai-Lei Zheng, Qiang Wang
