# Supplementary figures and images for: Pogostemon puerensis (Lamiaceae), a new species from Yunnan of China
Source: PhytoKeys. 2026 May 4;274:129–44. doi: 10.3897/phytokeys.274.186914 (PMC13161915; doi:10.3897/phytokeys.274.186914)

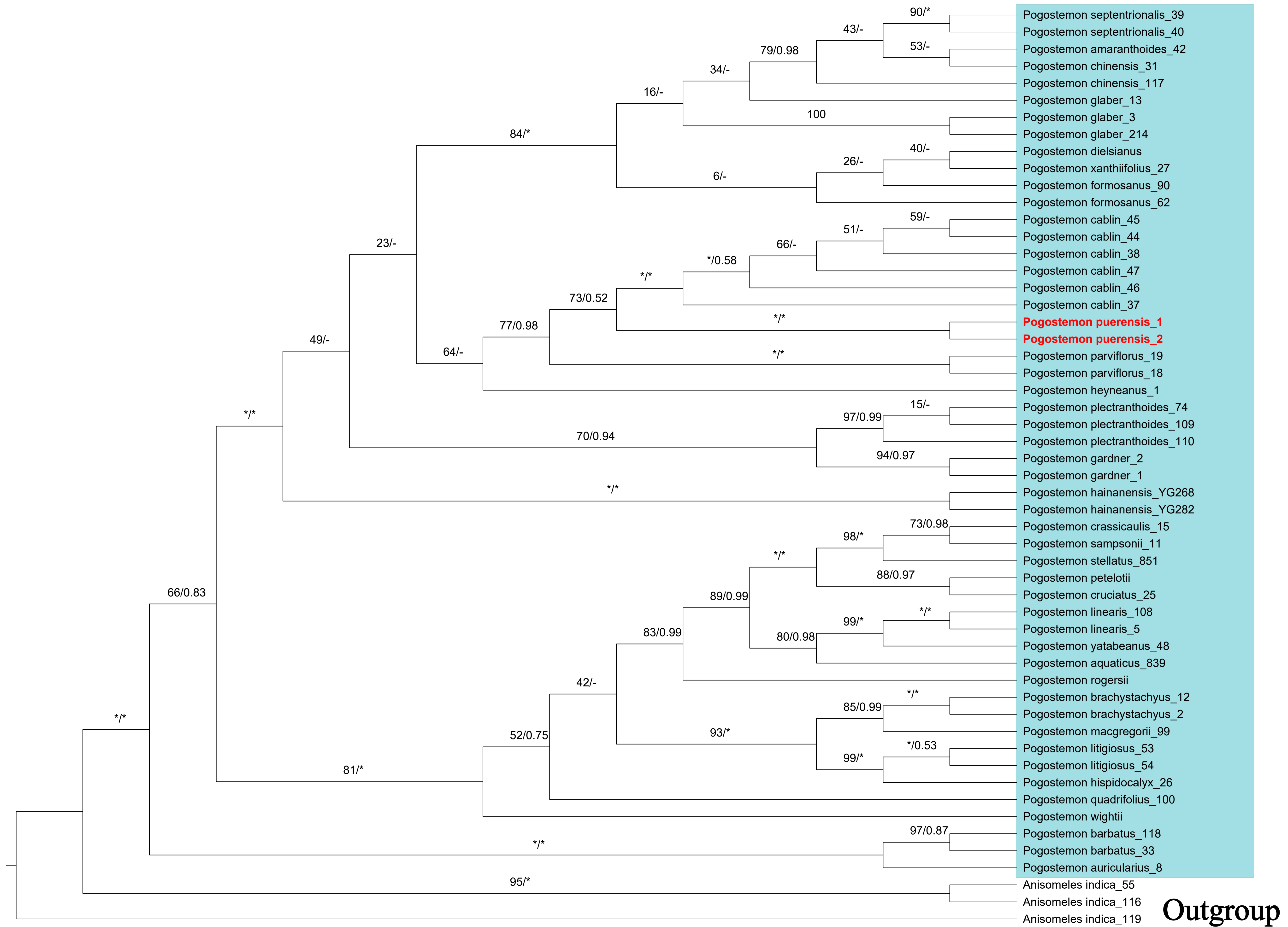

Outgroup

Supplement: Supplementary material 2 — Phylogenies of Pogostemon and related taxa based on the nrITS dataset, with Anisomeles as the sole outgroup, inferred using concatenated ML and BI analyses [file phytokeys-274-129_article-186914__-s002.pdf]
